# Topical prevention from high energy visible light-induced pigmentation by 2-mercaptonicotinoyl glycine, but not by ascorbic acid antioxidant: 2 randomized controlled trials

**DOI:** 10.3389/fphar.2025.1651068

**Published:** 2025-10-09

**Authors:** Virginie Piffaut, Romain De Dormael, Jean-Philippe Belaidi, Laudine Bertrand, Thierry Passeron, Françoise Bernerd, Claire Marionnet

**Affiliations:** ^1^ L’Oreal Research and Innovation, Aulnay-sous-Bois, France; ^2^ L’Oreal Research and Innovation, Saint-Ouen, France; ^3^ Department of Dermatology, CHU Nice, Université Côte D’Azur, Nice, France; ^4^ INSERM U1065, Centre Méditerranéen de Médecine Moléculaire, Université Côte D’Azur, Nice, France

**Keywords:** pigmentation, hyperpigmentation, visible light, solar light, high energy visible light, sunscreen, photoprotection, pigmentary disorders

## Abstract

**Introduction:**

Hyperpigmentation and pigmentary disorders are major clinical consequences of sun exposure. While UV radiation is a known contributor, visible light (VL), particularly High Energy Visible (HEV) light (400–450 nm), also induces long-lasting pigmentation in melanocompetent individuals (Fitzpatrick Phototype III and above), and can worsen pigmentary disorders. Therefore, photoprotection in this wavelengths range is recommended to prevent worsening of hyperpigmentation issues. Efficient solutions rely on the use of pigments, absorbing and diffusing VL. However, tint and opacity of these products may limit their use by consumers and patients. The search for actives preventing VL-induced pigmentation is therefore of interest. This work aimed at assessing 2 non tinted biological actives to counteract HEV-induced pigmentation.

**Material and Methods:**

Two very potent inhibitors of UV-induced pigmentation, ascorbic acid 7% (powerful antioxidant) and 2-mercaptonicotinoyl glycine (2-MNG, 0.5 or 1%, melanogenesis inhibitor), were assessed in 2 controlled randomized clinical trials (registered under the identification numbers NCT06945393 and NCT06937515 on ClinicalTrials.gov), including in total, 58 individuals with Fitzpatrick Phototype III or IV. Delineated areas on the subjects back, topically treated or not by the product, were exposed to HEV once a day for 4 days. The product was applied before, during and 5 weeks after HEV exposure. Pigmentation was assessed using chromametry and visual scoring throughout the studies.

**Results:**

While ascorbic acid did not exhibit any efficacy versus its vehicle in limiting skin hyperpigmentation induced by HEV, the use of 2-MNG (0.5 or 1%) led to an early significant decrease in HEV-induced pigmentation, which was sustained until the end of the study, as evidenced by colorimetry and significantly scored by visual assessment. Moreover, a 2-MNG dose effect could be evidenced at early time points.

**Conclusion:**

2-MNG represents an efficient and transparent alternative solution to pigments for the mitigation of HEV worsening of hyperpigmentation issues. This opens up perspectives for its use as a complement to UV protection afforded by sun filters.

## 1 Introduction

Hyperpigmentary disorders, such as melasma, post-inflammatory hyperpigmentation, and actinic lentigos, can affect individuals of all ages and skin types. Quality of life of affected individuals can be greatly altered, including psychological and sociological impacts (Wang et al., 2023). Internal (e.g., inflammation, hormonal changes) and external factors (e.g., sun exposure) can trigger and/or worsen hyperpigmentation (Passeron et al., 2020). Sun protection is then crucial to prevent the development and minimize recurrence of these disorders (Passeron et al., 2021).

In solar spectrum, ultraviolet (UV), including UVB (290–320 nm) and UVA (320–400 nm) rays, and visible light (VL, 400–780 nm) are both involved in pigmentary responses. UVB can activate melanogenesis, mostly via DNA damage response inducing accumulation of P53 and activation of the MITF transcriptional pathway (Cui et al., 2007); while UVA are mostly responsible for immediate and persistent pigment darkening (IPD/PPD) phenomena, which would result at least in part from melanin precursors oxidation following reactive oxygen species (ROS) production induced by UVA (Kollias and Bykowski, 1999; Marionnet et al., 2017; Ravnbak et al., 2009; Ravnbak and Wulf, 2007; Sklar et al., 2013).

VL is less energetic than UV rays but represents ∼43% of solar light received on earth (as compared to UV rays which represent 5%) and is a significant contributor to hyperpigmentation, inducing IPD/PPD (Kollias and Baqer, 1984; Pathak et al., 1962; Rosen et al., 1990) and a long-lasting hyperpigmentation restricted, in physiological conditions, to darker skin phototypes (skin types III to VI) (Mahmoud et al., 2010; Ramasubramaniam et al., 2011).

In VL, the highest energy band (400–450 nm) named high energy visible light (HEV) is the strongest contributor to VL-induced pigmentation, as shown using monochromatic sources, light emitting diodes (LED) or solar simulation (Duteil et al., 2014; Marionnet et al., 2023). By studying *in vivo* the impact of different VL wavelengths ranges, it was estimated that HEV accounted for 47% of VL-induced pigmentation at 24 h post exposure (Marionnet et al., 2023). Beneath this long lasting HEV-induced pigmentation, a mechanism of action leading to melanogenesis has been recently discovered. It implicates the blue light photoreceptor Opsin3 (OPN3), expressed at the surface of human melanocytes. Exposure of melanocytes to blue light can be sensed by OPN3, activating in a calcium dependent manner CAMKII, followed by CREB and p38 leading to MITF phosphorylation and the increase of key melanogenesis enzymes, such as tyrosinase and dopachrome tautomerase (Regazzetti et al., 2018). These results were confirmed by Yu et al., who also emphasized the role of TRPV1 which mediated this effect and the inhibition of clusterin (CLU), thus contributing to melanogenesis. Moreover, blue light exposure can decrease autophagy flux, suppressing melanosome degradation, further enhancing pigmentation (Yu et al., 2025).

VL, and especially HEV, is also responsible for ROS generation and subsequent oxidative stress (Opländer et al., 2011; Lawrence et al., 2018; Liebel et al., 2012; Mann et al., 2020). These VL or HEV-induced ROS could be also involved in skin darkening, although the impact of ROS in HEV-induced pigmentation was not evidenced (Regazzetti et al., 2018). Given the strong contribution of solar VL to hyperpigmentation, it is mandatory to afford photoprotection against this wavelengths range. Today tinted sunscreens which contain pigments (e.g., pigmentary titanium dioxide, yellow, red and black iron oxides) that absorb and diffuse VL, can provide an efficient protection against VL-induced pigmentation (Duteil et al., 2022; Duteil et al., 2017; Lyons et al., 2021; Renoux et al., 2025). However, the tint, coverage and opacity of these products may limit their use, especially for individuals with skin of color who encounter difficulties in finding matching shades in tinted sunscreens (De La Garza et al., 2022).

To overcome these issues, it appears important to find actives which could prevent pigmentation induced by VL, opening a way to non or less tinted products. Although several well-known and commonly used molecules, including tyrosinase inhibitors and antioxidants, are efficiently limiting UV-induced pigmentation, there is a lack for agents preventing or reducing VL or HEV-induced pigmentation.

In this work we aimed at testing 2 molecules to counteract HEV-induced pigmentation in 2 controlled randomized clinical trials, including in total 58 individuals with Fitzpatrick skin phototype (FSPT) III-IV.

Based on HEV potential in activating melanogenesis and inducing ROS, the efficacy against HEV-induced pigmentation of 2-mercaptonicotinoyl glycine (2-MNG, trademark Melasyl^TM^) as melanogenesis inhibitor (Sextius et al., 2024) and of ascorbic acid as strong antioxidant, was tested. These agents have already shown their efficiency in reducing UV-induced skin pigmentation. Notably, 2-MNG exhibited a strong, rapid and long-lasting efficacy against pigmentation induced by UV (UVA + UVB) (Al-Niaimi and Chiang, 2017; De Dormael et al., 2019; de Dormael et al., 2024). Among 14 anti-pigmenting and depigmenting agents, both molecules (2-MNG 0.5% and ascorbic acid 7%) were the most potent to inhibit UV-induced pigmentation, as shown in a Bayesian network meta-analysis (Muller et al., 2024).

## 2 Materials and methods

Two randomized, double-blind, intraindividual clinical trials (Study 1 and Study 2) were conducted independently on European volunteers.

In Study 1, the effect of ascorbic acid 7% on HEV-induced skin pigmentation was assessed versus vehicle at a single investigational site in Romania, from 29^th^ of October 2021 to 06^th^ of May 2022.

In Study 2, the effect of 2-MNG 1% or 0.5% on HEV-induced skin pigmentation was assessed versus vehicle at a single investigational site in Romania, from 27th of March 2023 to 1st September 2023.

2-MNG was patented (WO 2017/102349A1). 2-MNG toxicological profile has been determined using *in vitro* validated methods. Especially with respect to local tolerance, 2-MNG showed no skin or eye irritation potential, or skin sensitization potential, or phototoxic potential. Photostability of 2-MNG in formula is assessed under simulated solar outdoor radiation for all types of cosmetics depending on the use of the product. The good skin tolerability of the test substance in formulation has been confirmed during repeated applications in other dedicated human studies (on non-exposed or UV-exposed skin) (De Dormael et al., 2024). Full protocols and Clinical study reports are available at L’Oréal Research Center Aulnay-sous-Bois, 1 avenue Eugène Schueller; France.

### 2.1 Ethical statements

These studies respected local legal requirements and were performed according to the principles of the Declaration of Helsinki. The principal investigator was the site dermatologist, responsible for assuring compliance with applicable regulations, for the oversight of the study and the informed consent process. All subjects provided informed consent before any study procedure. The study followed ICH-GCP for the clinical part, including the data management and the statistical analyses. In Romania, the cosmetic studies don’t have to be approved by Ministry of Health nether than any Ethic Committee. Each subject provided a written informed consent prior to any procedure. Studies are registered under the ClinicalTrials.gov site with NCT06945393 for Study 1 and NCT06937515 for Study 2.

### 2.2 Demography and inclusion criteria

In Study 1, 35 healthy European female (n = 18) and male (n = 17) subjects were randomized and 30 completed the study. 2 subjects withdrawn their consent, 2 were excluded due to erythema on the investigational zones, and one was excluded due to COVID-19 infection. Inclusion criteria were: 18–50 y.o, with Fitzpatrick skin phototypes (FSPT) III or IV and individual typology angle (ITA°) ranging from 18° to 32°. The included panel was aged from 18 to 50 y.o (mean 34.3 y.o), with FSPT III (n = 20) and IV (n = 15), and the ITA° ranging from 21° to 30° (mean 28°).

**FIGURE 1:**
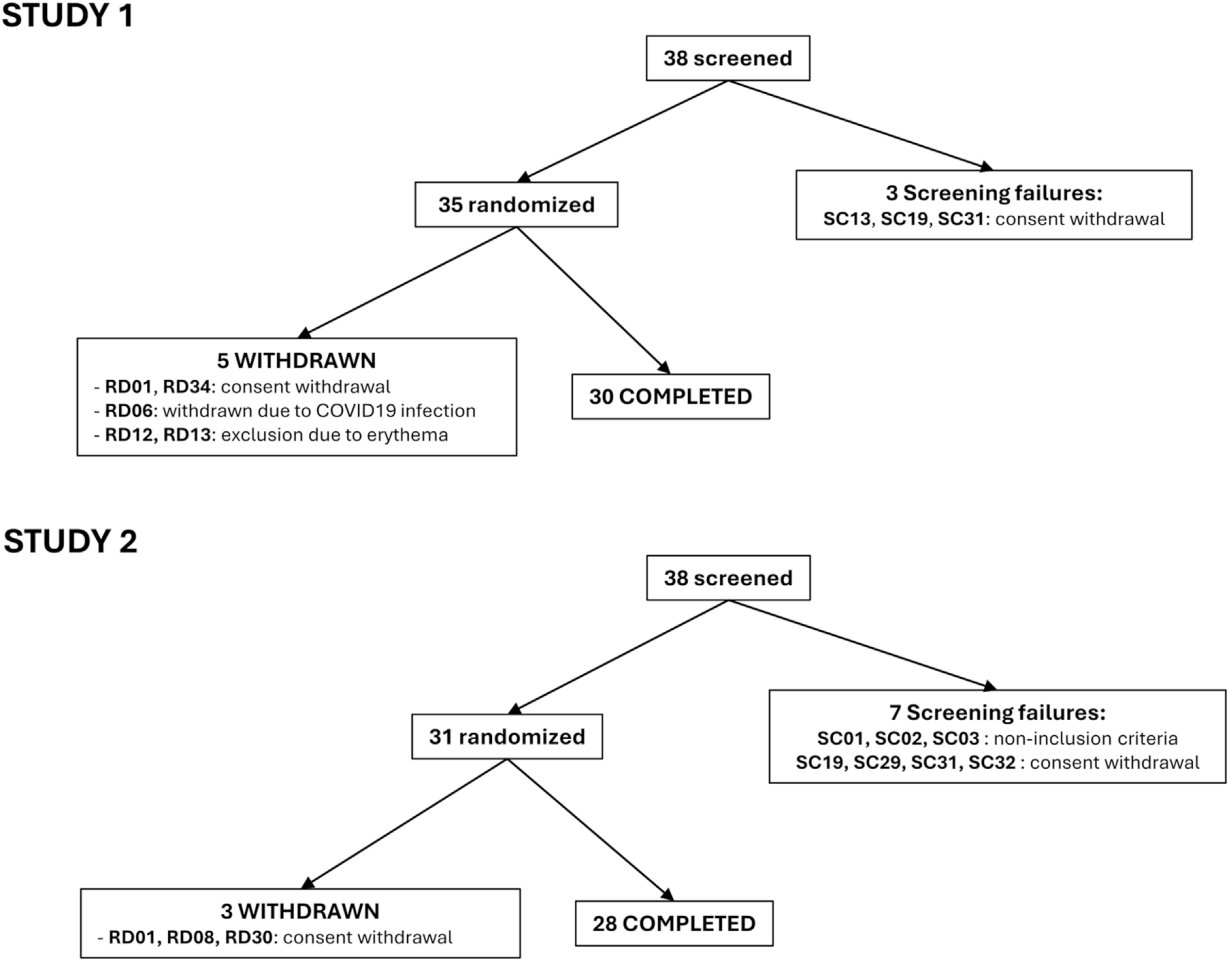
Flow chart for Study 1 and Study 2.

In Study 2, 31 healthy European, female (n = 20) or male (n = 11) subjects were randomized and 28 completed the study. 3 subjects withdrawn their consent. The included panel was aged from 21 to 49 y.o (mean 38.6 y.o), with FSPT III (n = 19) and IV (n = 12) and ITA° ranging from 19° to 33° (mean 28°) (Table 1; Figure 1).

**TABLE 1:** Demographic data at baseline for Study 1 and Study 2. F, Female (gender at birth); M, Male (gender at birth); yo, year old.

Study #	Number of randomized subjects	Gender (n)	Fitzpatrick skin phototype (n)	Age		ITA°	
				Mean	Min- Max	Mean	Min-Max
Study 1	35	F (18); M (17)	III (20); IV (15)	34.3 yo	18–50 yo	28°	21°–30°
Study 2	31	F (20); M (11)	III (19); IV (12)	38.6 yo	21–49 yo	28°	19°–33°

ITA° is derived from the L*a*b* color space (CIE, 1976) using the formula: ITA° = Arctan (L* − 50)/b*) 180/π.

For both studies, subjects were requested not to expose themselves under solar or artificial sources during the entire study duration and were excluded if they had tan marks, freckles, nevi or hairs on the back, or any history of abnormal response to the sun.

### 2.3 Assignment and masking

The sample size estimation was based on previous studies assessing prevention of UV-induced skin pigmentation by anti-pigmenting and depigmenting agents, including 2-MNG and ascorbic acid 7% (de Dormael et al., 2024; De Dormael et al., 2019) or studies assessing HEV-induced pigmentation (Marionnet et al., 2023).

At Day 1 (D1, Baseline), all subjects fulfilling the inclusion criteria were assigned a randomization number (provided by the sponsor via eCRF) in chronological order of inclusion. No number was omitted or skipped. The randomization list was generated by a computer process before the study by the sponsor and integrated into the eCRF. The investigator was supplied with sealed envelopes provided by the sponsor for each subject number, with information of the formula assignment to each investigational zone. The corresponding envelope was only opened in case of safety concerns (local intolerance, adverse event, serious adverse event).

### 2.4 Treatments

In Study 1, 3 areas (3 × 3 cm: 9 cm^2^) were delineated on the back of the subjects, including 2 treated zones and a non-treated zone. The position of each product on the back of a volunteer was randomly allocated according to a preestablished randomization by incomplete blocks, providing a blind distribution on the application areas. Each subject received ascorbic acid 7% and its vehicle, formulated as previously described (De Dormael et al., 2019). One area was not treated.

In Study 2, 4 areas (3 × 3 cm: 9 cm^2^) were delineated on the back of the subjects, including 3 treated zones and a non-treated zone. As in Study 1, the position of each product on the back of a volunteer was randomly allocated. Each subject received: 2-MNG 1%; 2-MNG 0.5% and vehicle, formulated as previously described (de Dormael et al., 2024). One area was not treated.

For both studies, products were applied at a dose of 4 mg/cm^2^ by a trained technician with the following design: during the first week, twice a day, 5 days per week; during the second week, thrice a day, 4 days per week from D8 to D11 (1 h before, immediately and 20 min after HEV exposure) and twice a day on D12; during the third week, twice a day, 5 days per week; from week 4 to week 6, once a day, 5 days per week; and during week 7, once a day, 4 days per week, as described in Table 2.

**TABLE 2:** Clinical protocol including product applications, HEV exposure and pigmentation assessments. Products were applied at the investigational center, 5 days a week for 7 weeks (except on day 47). Depending on the week, products were applied from once daily to thrice daily. HEV exposure was once a day, at week 2, on days 8, 9, 10 and 11. Pigmentation assessements were performed on indicated days, before product application.

Week/day	Product application	HEV exposure	Assessments
Week 1			
D1	Twice a day	No	Yes
D2	Twice a day	No	No
D3	Twice a day	No	No
D4	Twice a day	No	No
D5	Twice a day	No	No
Week 2			
D8	Thrice a day (before, immediately after and at least 4 h after HEV exposure)	Yes	Yes
D9		Yes	Yes
D10		Yes	Yes
D11		Yes	Yes
D12	Twice a day	No	Yes
Week 3 (D15-19)	Twice a day	No	On D15, D19
Week 4 (D22-D26)	Once a day	No	On D22, D26
Week 5 (D29-D33)	Once a day	No	On D29, D33
Week 6 (D36-D40)	Once a day	No	On D36, D40
Week 7 (D43-D47)	Once a day (except on D47)	No	On D43, D47

### 2.5 HEV exposure

**FIGURE 2:**
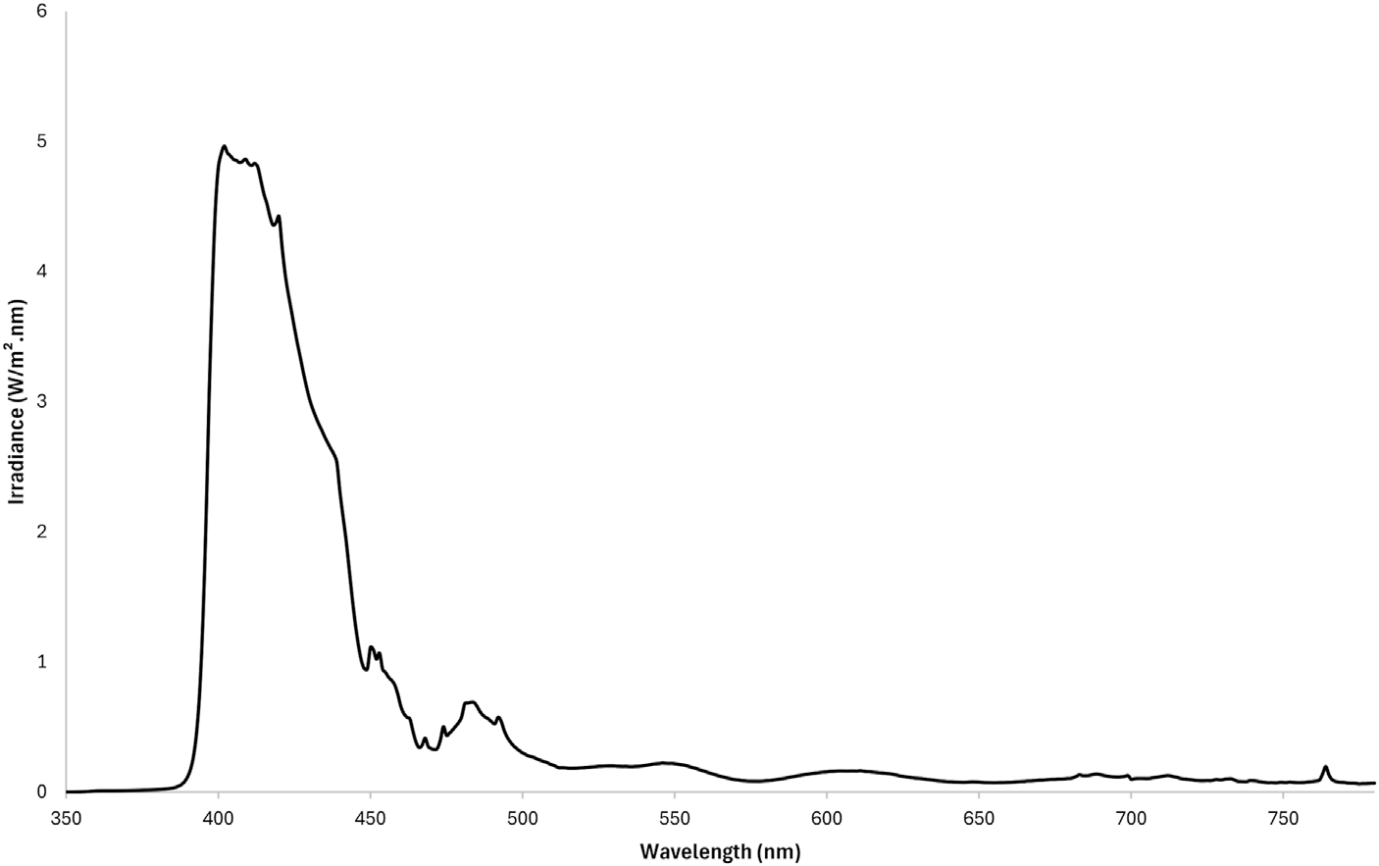
HEV emission spectrum used in Study 1 and Study 2. For both studies, the same Oriel 1,600 W solar simulators were equipped with a dichroic mirror, a WG360CM-fil0.6 filter and a multilayer filter designed to obtain a cut-off at 400 nm, leading to HEV spectrum (390–450 nm).

HEV spectrum was delivered by a solar simulator (ORIEL 1600-W lamp, Oriel Instruments, Stratford, CT, United States) equipped with a dichroic mirror, a WG360CM-fil0.6 filter (Monaderm, Monaco) and a thin multilayer filter made of alternating layers of TiO2 and SiO2 (TOA Optical Technologies LTD., Tokyo, Japan) designed to obtain a cut-off at 400 nm leading to HEV spectrum (Marionnet et al., 2023) (Figure 2).

The investigational areas were exposed to 35J/cm^2^ HEV (as measured between 400 and 450 nm) on D8, D9, D10 and D11 (Table 2). This dose was described as adequate to reveal enough pigmentation for this protocol (Marionnet et al., 2023).

### 2.6 Assessments

Colorimetric measurements and visual evaluations of skin pigmentation were assessed at baseline, before the first product application and before the first HEV exposure, 24 h after each HEV exposure, 4 and 8 days after the last HEV exposure and twice weekly until week 7. Assessments were performed on each HEV-exposed and treated, unexposed and treated, and on untreated and unexposed sites (Table 2).

Colorimetric measurements were performed using a Chromameter^®^ Minolta CR400 (Konika Minolta, Tokyo, Japan) with L*a*b* color system (CIE lab, 1976).

The efficacy of products on HEV-induced skin pigmentation was expressed as the difference in ΔITA and Δ*E* compared to its vehicle with Δ*E* defined in 1976 by the international Commission of illumination (CIE) as follows:

Δ*E* = √(Δ*L* ^2^ + Δ*a* ^2^ + Δ*b* ^2^), where Δ represents the difference between exposed and unexposed treated areas.

Visual scoring was performed by a trained technician for pigmentation on a scale from 0 (No pigmentation) to 13 (Pronounced brown +) under a daylight ceiling D65.

Safety was assessed throughout as adverse event reporting, including local tolerance.

### 2.7 Statistical analysis

Linear mixed models were used to compare measurements between different time points and exposure conditions. These models included subject and time as random effects, and baseline measurement, exposure condition, time, and the interaction between exposure condition and time as fixed effects. An AR (1) covariance structure was implemented to account for the correlation of repeated measurements within subjects, addressing the longitudinal nature of the data. All statistical tests were two-sided and conducted with a Type I error rate (alpha) of 0.05. The Benjamini–Hochberg procedure was employed to control the false discovery rate associated with multiple comparisons.

Prior to any analysis, the homogeneity of treated and untreated unexposed skin areas was assessed to confirm that the products had no effect on unexposed skin. Statistical computations were performed using the R statistical programming language.

## 3 Results

### 3.1 Study 1

In the Study 1, the efficacy of ascorbic acid 7% against HEV-induced pigmentation was assessed. 30 European subjects with FSPT III or IV, and mean ITA° of 28°, completed the study.

**FIGURE 3:**
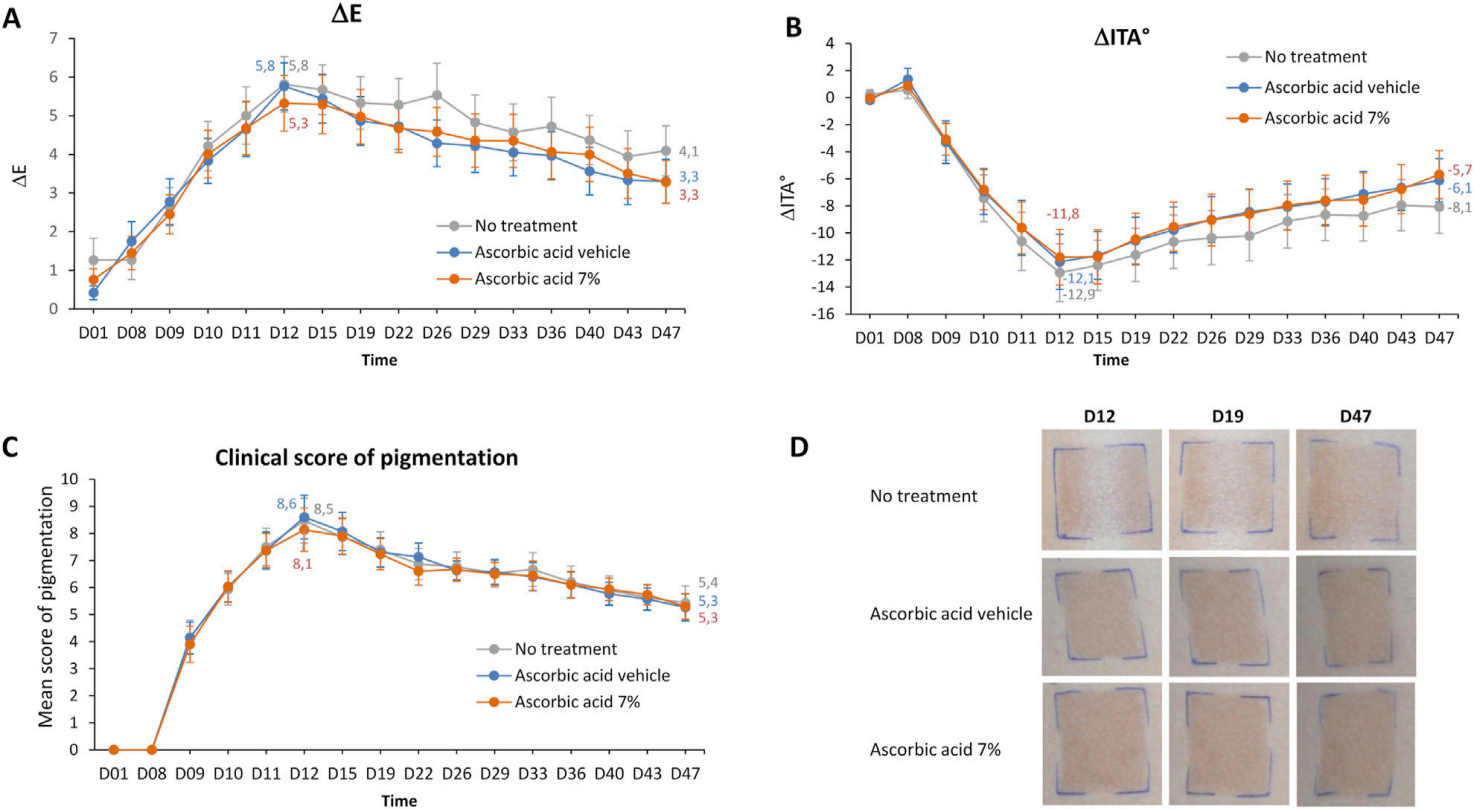
Study 1 – Pigmentation assessment after exposure to HEV spectrum of skin area treated or not by ascorbic acid 7% or its vehicle. 4 mg/cm^2^ of product was applied at several time points per zone onto the back skin of 30 volunteers the week before, during and the 5 weeks after the 4 HEV exposures (4 × 35 J/cm^2^). Colorimetric parameters (L*, a*, b*) were measured at D1, before each HEV exposure and at several time points after exposure. Difference in skin pigmentation ΔE **(A)** and skin color ΔITA° **(B)** between HEV-exposed and adjacent-unexposed zones were calculated for each zone. In parallel, a visual pigmentation scoring was performed using a 13-point scale grading from absence (0) to pronounced brown pigmentation (13). Mean scores of pigmentation were plotted **(C)**. Representative photographs at D12; D19, and D47 is shown **(D)**. Values are expressed as means ± 95% CI. Numerical values are indicated for D12 (maximum level of pigmentation) and D47 (last day of the study). CI, confidence interval; h, hour; ITA, individual typology angle; D, day.

A significant increase in skin pigmentation was detected from D9 (24 h after the first HEV exposure) to D12 (24 h after the 4th HEV exposure), reaching its maximum at the latter time point (ΔE = 5.8, ΔITA = −12.9 and mean pigmentation score = 8.5 for the non-treated exposed zone vs. its adjacent non-exposed zone). The pigmentation then slowly decreased until D47. On this last day of the study, the level of pigmentation was still higher than the baseline pigmentation level at D1 (ΔE = 4.1, ΔITA = −8.1, mean pigmentation score = 5.4) (Figures 3A–C). Treatment with the vehicle of ascorbic acid 7% did not significantly change the level of pigmentation compared to the non-treated zone, as assessed using ΔE, ΔITA or pigmentation score (Figures 3A–C).

No significant differences in pigmentation level were found between ascorbic acid-treated and vehicle-treated zones, neither using chromametric assessment (Figures 3A,B; Supplementary Figure S1) nor by visual pigmentation scoring (Figure 3C). This indicated that the applications of ascorbic acid 7% did not prevent HEV-induced pigmentation, as observed on the representative photographs (Figure 3D).

### 3.2 Study 2

In Study 2, 2-MNG, at 0.5% and 1%, and its vehicle were tested. The subjects were recruited according to similar criteria of inclusion and submitted to the same protocol than in Study 1. In all, 28 European subjects, with FSPT III and IV and mean ITA value of 29° completed the Study 2. In the present clinical study, 2-MNG presented a very good tolerability on nonexposed and HEV-exposed skin irrespective of its concentration, without any adverse event or phototoxic/photoallergic reaction.

Like in Study 1, skin pigmentation was increased from D9 to D12, reaching its maximal level (ΔE = 5.6, ΔITA = −11.7), and decreased until D47, while not reaching its baseline level. The profile and level of skin pigmentation were similar between Study 1 and Study 2 (Supplementary Figure S2).

The vehicle of 2-MNG had no impact on HEV-induced pigmentation, as compared to non-treated exposed zone, and measured using ΔE, ΔITA or visually scored (Supplementary Figure S3).

**FIGURE 4:**
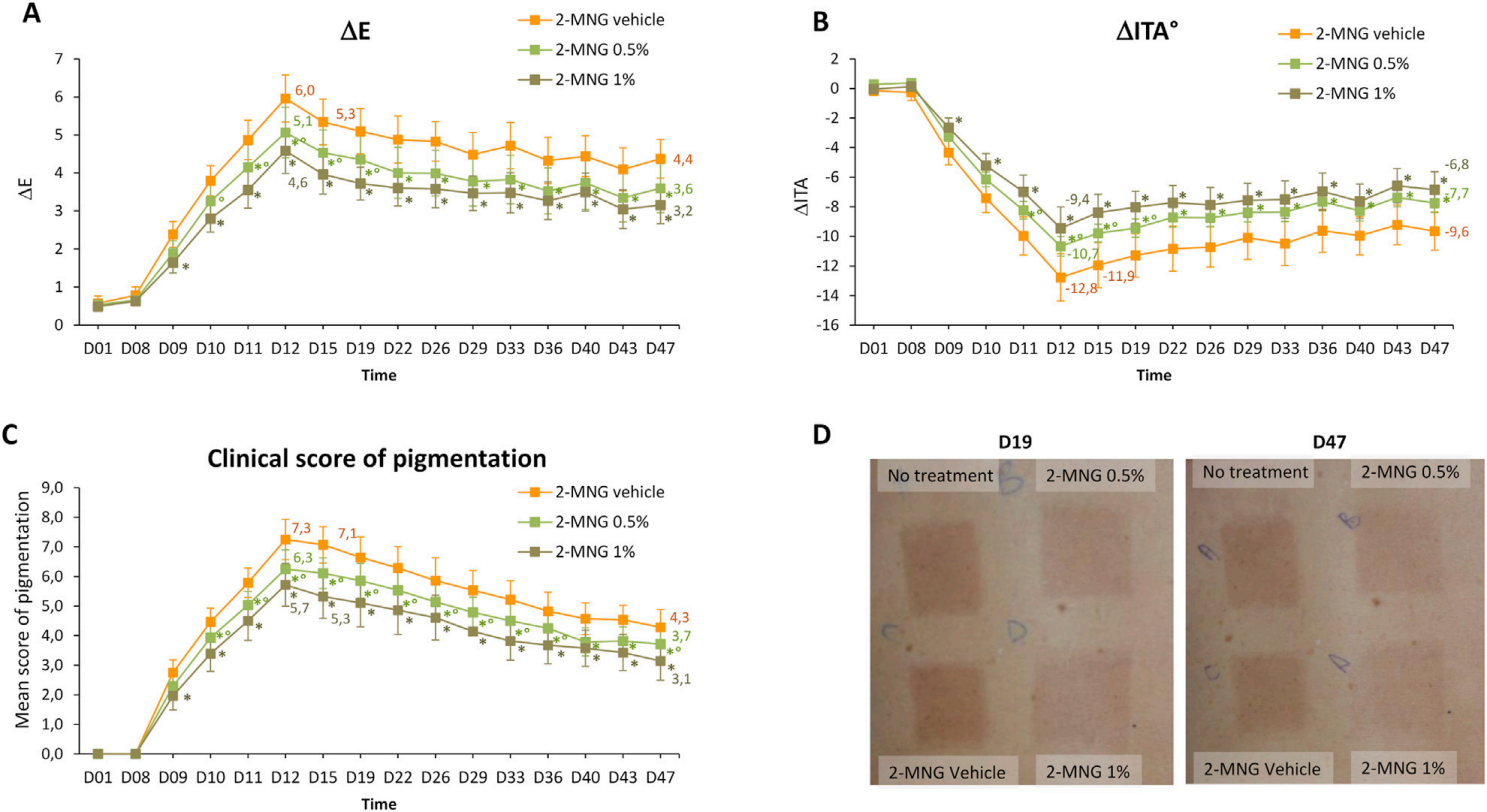
Study 2 - Pigmentation assessment after exposure to HEV spectrum of skin area treated or not by 2-MNG (0.5% or 1%) or by its vehicle. 4 mg/cm^2^ of product was applied at several time points per zone onto the back skin of 28 volunteers the week before, during and the 5 weeks after the 4 HEV exposures (4 × 35 J/cm^2^). Colorimetric parameters (L*, a*, b*) were measured at D1, before each HEV exposure and at several time points after exposure. Difference in skin pigmentation ΔE **(A)** and skin color ΔITA° **(B)** between HEV-exposed and adjacent-unexposed zones were calculated for each zone. In parallel, a visual pigmentation scoring was performed using a 13-point scale grading from absence (0) to pronounced brown pigmentation (13). Mean scores of pigmentation were plotted **(C)**. Representative photographs at D19 and D47 are shown **(D)**. Values are expressed as means ± 95% CI. *, significant difference compared to vehicle-treated zone; °, significant difference compared to 2-MNG 1%-treated zone. CI, confidence interval; h, hour; ITA, individual typology angle; D, day.

Figure 4 shows the comparison of pigmentation on 2-MNG- and vehicle-treated zones (colorimetric variables ΔL*, Δa*, Δb* are detailed in Supplementary Figure S4). The topical application of 2-MNG 1% significantly reduced HEV-induced pigmentation compared to the use of 2-MNG vehicle, as early as D9 (i.e. 24 h after the first HEV exposure), and at all time points until D47, as assessed using ΔE and ΔITA (Figures 4A,B). At D12, at the maximum of HEV-induced pigmentation, a 1.4 difference of ΔE values between 2-MNG- and vehicle-treated zones was measured, (for ΔITA the difference was 3.3). At D47, i.e. 5 weeks post exposure, ΔE difference between 2-MNG 1%- and vehicle-treated zones was still 1.2, ΔITA difference was 2.8 (Figures 4A,B). It is considered that a ΔE difference above 0.7 and a ΔITA difference above 2 can be perceptible by a trained assessor. This was confirmed by visual scoring of pigmentation by the expert: from D9 to D47, pigmentation mean score was significantly lower on the 2-MNG 1%-treated zone compared to vehicle treated zone, with a maximum difference of 1.8 scored at D15.

The topical application of 2-MNG at 0.5% also enabled a significant decrease of HEV-induced pigmentation compared to the vehicle use. This decrease appeared early (from D11) and was long-lasting (at all time points until D47) with regards to ΔE and ΔITA values (Figures 4A,B). It was visually detected from D10 and at all time points until D47 (Figure 4C). At D12, the difference of ΔE between 2-MNG 0.5% treated and vehicle-treated zones was 0.9, for ΔITA it was 2.1 (Figures 4A,B), corresponding to a 1 point difference in terms of visual scoring (Figure 4C).

The comparison of pigmentation between 2-MNG 1% and 0.5% revealed mild but significant differences at early time points, from D10 to D19 for ΔE (maximum difference of 0.6 at D19) and from D11 to D19 for ΔITA (maximum difference of 1.4 at D19), corresponding to a 0.75 difference value by visual scoring. This 2-MNG dose effect was not significant at later time points.

Photographs of the back of a representative subject illustrate the results of Study 2 (Figure 4D).

## 4 Discussion

In order to find alternative or additional solutions to tinted products containing pigments to reduce pigmentation induced by visible light, this work aimed at evaluating the efficacy of 2 molecules (ascorbic acid and 2-MNG) against HEV-induced pigmentation, in 2 randomized controlled trials.

### 4.1 The clinical protocol

The protocol used was adapted from the one testing anti-pigmenting or depigmenting agents after UV exposure (De Dormael et al., 2019; de Dormael et al., 2023). In the present work, the studies were carried out on phototypes III-IV individuals, with melanocompetent skin, who have been shown to experience skin pigmentation after visible light or HEV exposure (Duteil et al., 2017; Mahmoud et al., 2010). HEV light was used, with 4 HEV exposures (one per day) at a dose of 35 J/cm^2^. This HEV dose is physiologically relevant and can be received in ∼ 1h10 in Sao Paulo 15 December at noon or in ∼1 h 40 min in Paris 15 April at noon; and can induce a significant pigmentation (Marionnet et al., 2023). In Study 1 and Study 2, the 4 consecutive HEV exposures allowed a good amplitude in skin pigmentation, until the last day of the studies (i.e. 5 weeks after the last exposure). Moreover, evolution in time and level of pigmentation were similar between both studies, allowing us to compare their respective results. In these studies, the immediate pigmentation (IPD) was not assessed. At low dose IPD is reversible, and at higher dose it becomes persistent (PPD detected 2–24 h post exposure), the latter blending into long-lasting or delayed pigmentation (up to several weeks) (Sklar et al., 2013). We then wanted to focus on persistent and long-lasting pigmentation induced by repeated exposures, mimicking an everyday life situation, to evaluate the efficacy of the products.

### 4.2 Assessment of ascorbic acid antioxidant

Ascorbic acid is one of the most powerful skin antioxidant, neutralizing oxidative stress by a process of electron transfer and/or donation (Njus et al., 2020). It is an effective skin lightener and was shown to prevent UV-induced skin pigmentation, likely thanks to its anti-oxidative properties (Ando et al., 2007; Kameyama et al., 1996; Kumano et al., 1998; Ochiai et al., 2006). A recent meta-analysis including more than 700 healthy volunteers (Fitzpatrick STIII; ITA°28°–49°) clearly demonstrated its efficiency against pigmentation induced by repeated UVA + UVB exposures, especially during the exposure phase and 24 h after the last exposure. These time points rely on PPD, which is believed to result from oxidation of melanin precursors, that would be counteracted by the anti-oxidative properties of ascorbic acid (De Dormael et al., 2019).

Here, using the same clinical protocol (same number of applications at the same doses of ascorbic acid, same time course and assessments) and a population with similar constitutive skin pigmentation and age range, than in De Dormael et al. studies, we showed that the pigmentation induced by HEV exposure was not prevented by ascorbic acid 7%. This result highlights the complexity and diversity of mechanisms of action in skin pigmentation induction by different wavelengths, as detailed in the introduction section.

Other antioxidants have been assessed in previous studies, either alone or in combination with sunscreens. In subjects with Fitzpatrick ST IV to VI exposed to VL + UVA, topical application of a blend of 3 antioxidants (Diethylhexyl syringylidene malonate, Vitamin E, Ascorbyl Palmitate) or of a sunscreen formula enriched with 5 antioxidants (Diethylhexyl syringylidene malonate, Vitamin E, Vitamin C, Licochalcone A, Glycyrrheztinic acid) could mitigate IPD, when compared to untreated zone. However, in line with our results, persistent pigmentation, measured 24 h after exposure (PPD) and long-lasting pigmentation, measured 7 days post exposure, were not significantly prevented as compared to untreated skin area (Lyons et al., 2022; Ruvolo et al., 2022).

Moreover, with regards to the biological mechanism described for HEV-induced pigmentation, i.e., activation of melanogenesis via OPN3 photoreceptor (Regazzetti et al., 2018; Yu et al., 2025), it was shown that the use of N-Acetyl Cystein (NAC) antioxidant did not prevent HEV-induced melanogenesis pathway in melanocytes *in vitro* (Regazzetti et al., 2018).

### 4.3 Assessment of the melanogenesis inhibitor 2-MNG

As HEV exposure can induce melanogenesis (Regazzetti et al., 2018; Yu et al., 2025), the efficacy of the novel melanogenesis inhibitor 2-MNG was also assessed against HEV-induced pigmentation. Its strong, rapid and long-lasting efficacy against pigmentation induced by UV (UVA + UVB) (de Dormael et al., 2024) ranked it first compared to 13 reference molecules, as evidenced in a recent meta-analysis (Muller et al., 2024).

Here, in addition to its potency against UV-induced pigmentation, we showed a novel property of 2-MNG, with a significant efficiency in reducing HEV-induced pigmentation, at levels which could be measured using colorimetry but also visualized and scored. The action of 2-MNG was early (24 h after the first HEV exposure), at its maximum level 24–96 h after the last exposure and remained persistent up to the last day of the study, in line with the potency of 2-MNG to bind to melanin precursors like dopaquinone, DHI quinone and DHICA quinone, preventing their integration into melanin pigments and effectively reducing both eumelanin and pheomelanin production (Sextius et al., 2024). The efficacy was shown for the 1% and 0.5% concentrations of 2-MNG, with a dose effect detected at early time points.

### 4.4 Limitations of the study

One limitation of the study relies on the restricted FSPT and ITA° range of the subjects. It could be of interest to evaluate the products on subjects with darker skin types.

In addition, as not only blue light but also green light or VL can induce pigmentation (Marionnet et al., 2023), it could be valuable to test the efficacy of the products under the latter wavelength ranges.

Since up today only tinted products showed efficacy against VL-induced pigmentation, another limitation of the study is that 2-MNG was not compared to a product containing pigments. As the efficacy is correlated to the amount of pigments (Duteil et al., 2022; Renoux et al., 2025), it would be worth to compare 2-MNG to several products with various levels of pigments.

Moreover, as sun pigmentation is induced by UV and visible light it would be valuable to evaluate sunscreen products containing actives and UV filters under a full solar simulated spectrum.

### 4.5 Conclusion

We showed that the topical use of the strong antioxidant ascorbic acid at a high concentration did not prevent either early, persistent or long-lasting pigmentation induced by HEV. Nevertheless, as the involvement of VL or HEV-induced ROS in skin darkening cannot be ruled out, it would be of interest to test other types of antioxidants, which could quench different radical species.

Importantly, we evidenced 2-MNG as the first topical biological active able to significantly and visibly reduce persistent and long-lasting HEV-induced pigmentation, with a rapid action, in complement to its previously evidenced efficacy against pigmentation induced by UV.

This represents an efficient complementary solution to pigments in sunscreen products for the mitigation of solar worsening of hyperpigmentation issues. Additionally, this transparent alternative addresses patients expectation for non-covering and non-colored products.

## Data Availability

The original contributions presented in the study are included in the article/Supplementary Material, further inquiries can be directed to the corresponding author.
