# Supplementary figures and images for: Topical prevention from high energy visible light-induced pigmentation by 2-mercaptonicotinoyl glycine, but not by ascorbic acid antioxidant: 2 randomized controlled trials
Source: Front Pharmacol. 2025 Oct 9;16:1651068. doi: 10.3389/fphar.2025.1651068 (PMC12546040; doi:10.3389/fphar.2025.1651068)

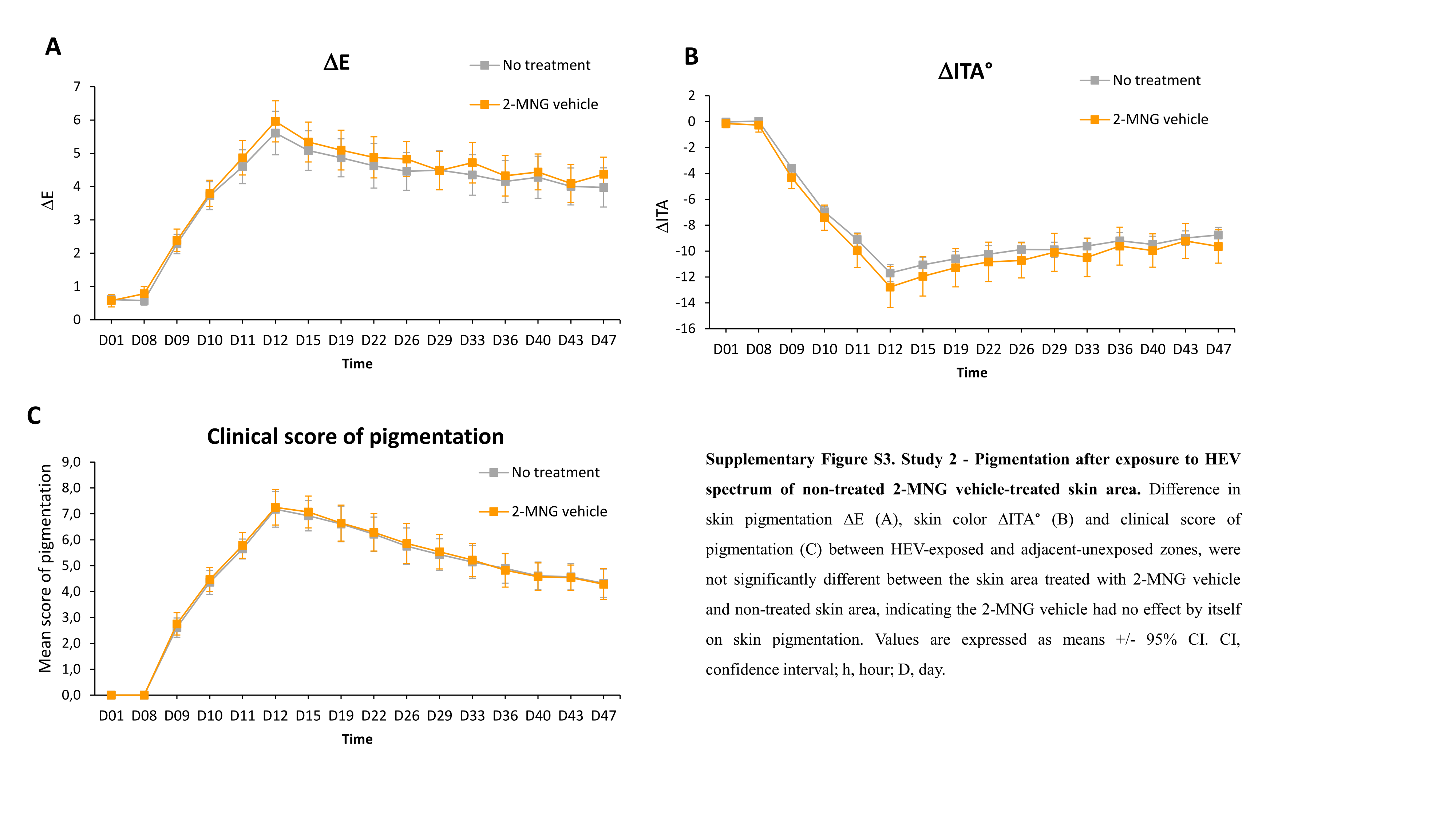

Supplement: Supplementary file 1 [file Image3.tiff]

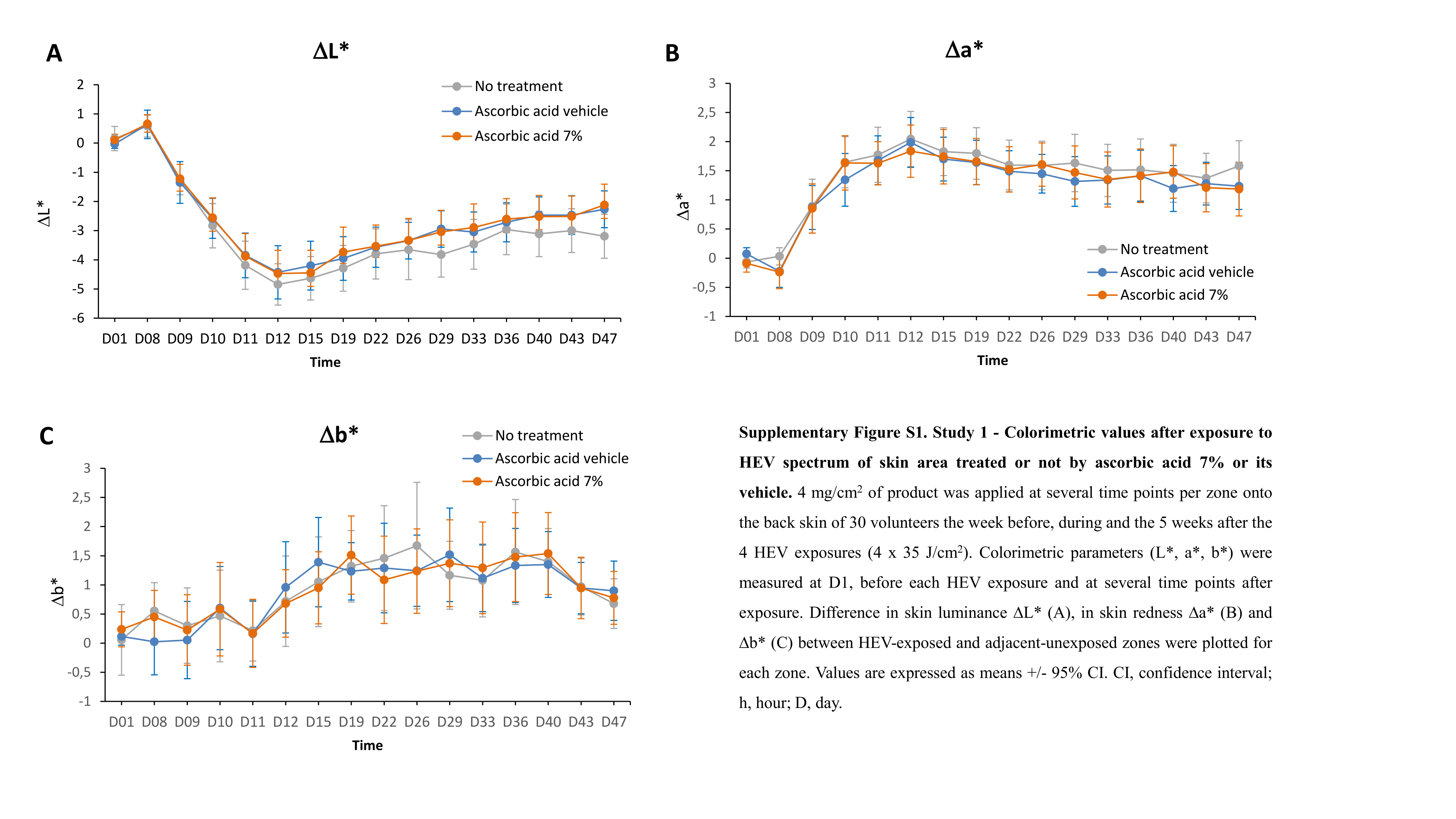

Supplement: Supplementary file 2 [file Image1.tiff]

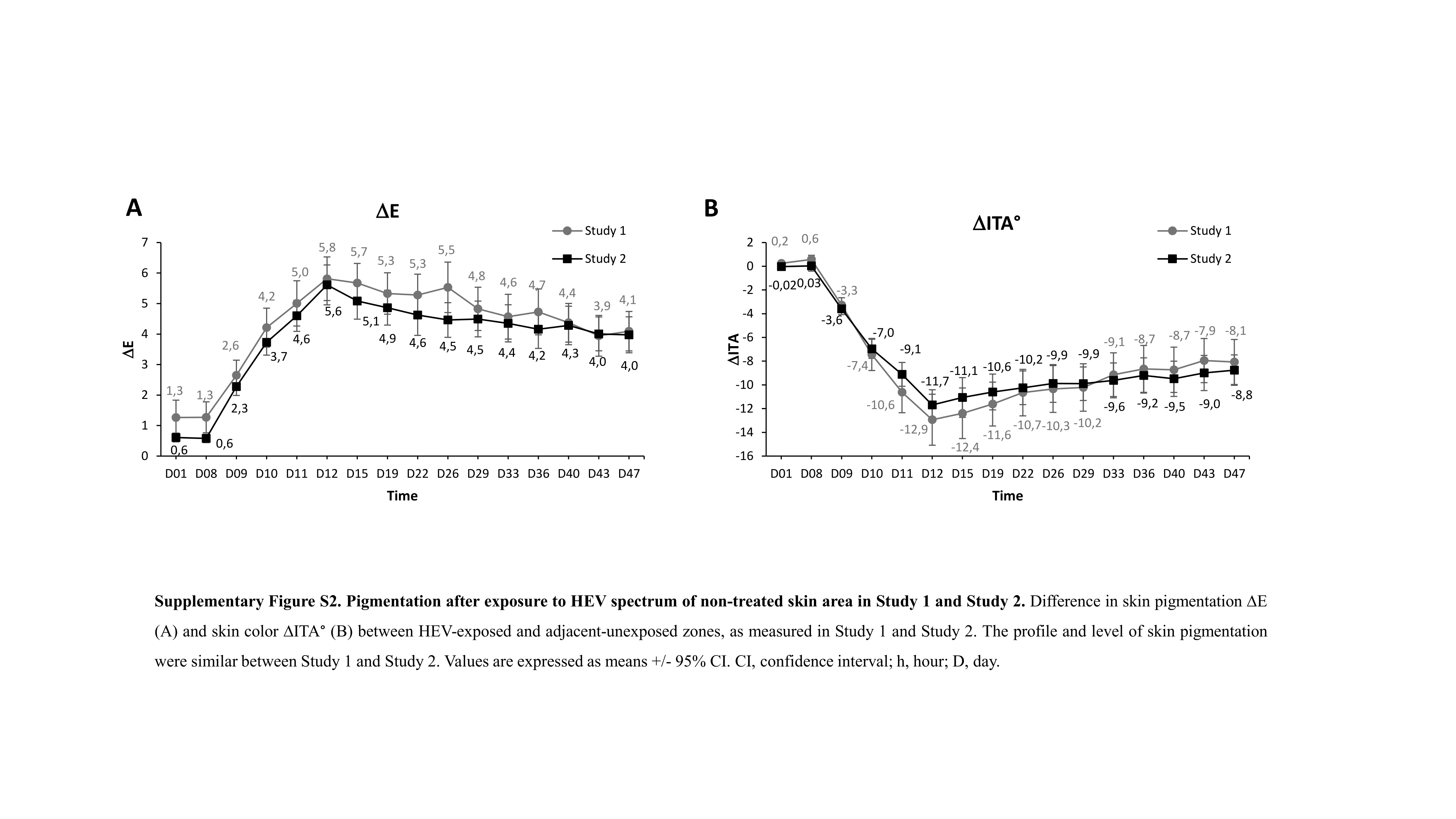

Supplement: Supplementary file 3 [file Image2.tiff]

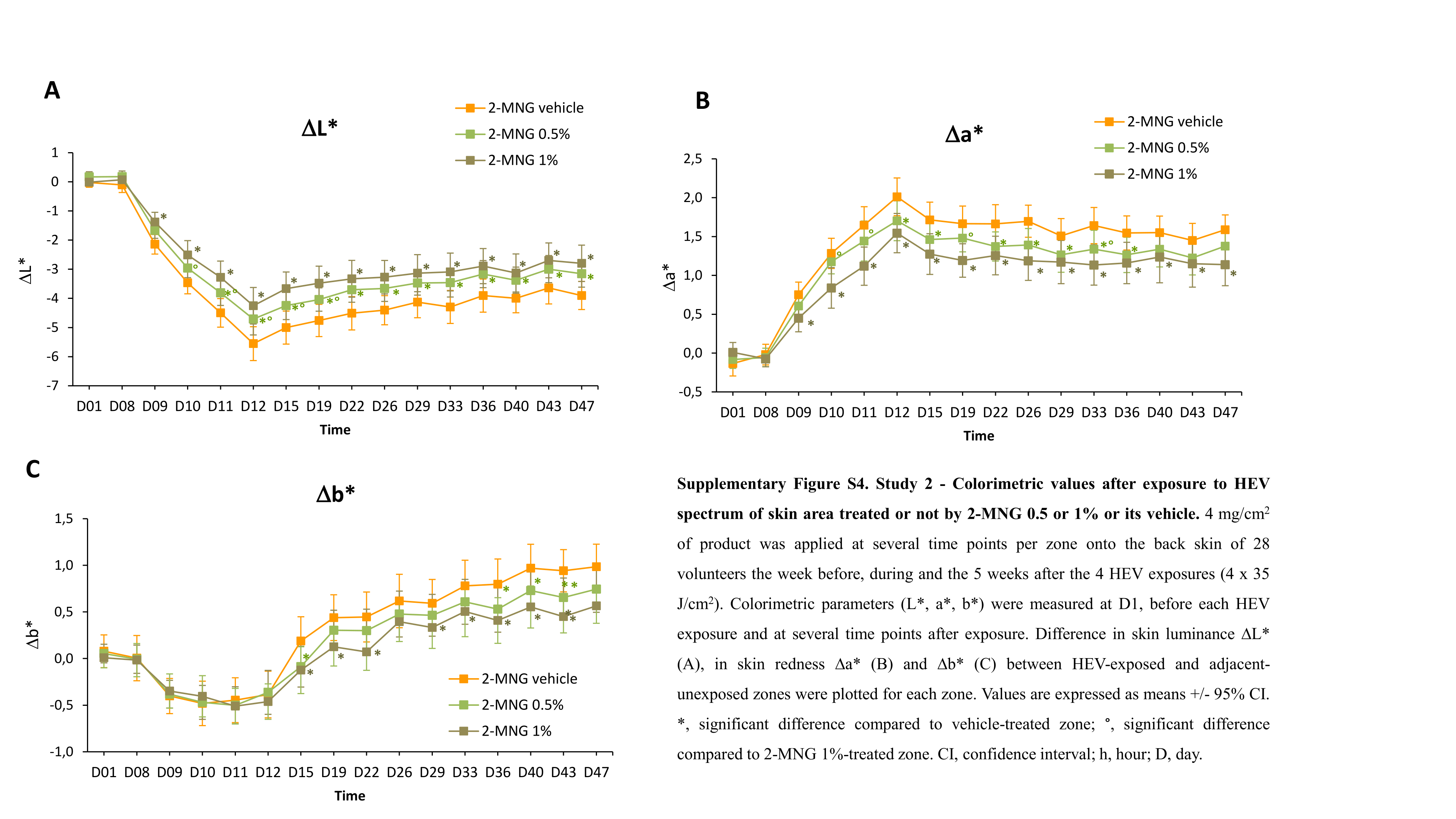

Supplement: Supplementary file 4 [file Image4.tiff]
